# Classical NF-κB Metabolically Reprograms Sarcoma Cells Through Regulation of Hexokinase 2

**DOI:** 10.3389/fonc.2018.00104

**Published:** 2018-04-11

**Authors:** Priya Londhe, Peter Y. Yu, Yuichi Ijiri, Katherine J. Ladner, Joelle M. Fenger, Cheryl London, Peter J. Houghton, Denis C. Guttridge

**Affiliations:** ^1^Department of Cancer Biology and Genetics, The Ohio State University, Columbus, OH, United States; ^2^Arthur G. James Comprehensive Cancer Center, The Ohio State University, Columbus, OH, United States; ^3^Medical Student Research Program, The Ohio State University, Columbus, OH, United States; ^4^Department of Veterinary Biosciences, College of Veterinary Medicine, The Ohio State University, Columbus, OH, United States; ^5^Cummings School of Veterinary Medicine, Tufts University, Grafton, MA, United States; ^6^Greehey Children’s Research Institute, University of Texas Health Science Center, San Antonio, TX, United States

**Keywords:** sarcoma, nuclear factor kappa B, metabolism, hexokinase, rhabdomyosarcoma, osteosarcoma

## Abstract

**Background:**

Metabolic reprogramming has emerged as a cancer hallmark, and one of the well-known cancer-associated metabolic alterations is the increase in the rate of glycolysis. Recent reports have shown that both the classical and alternative signaling pathways of nuclear factor κB (NF-κB) play important roles in controlling the metabolic profiles of normal cells and cancer cells. However, how these signaling pathways affect the metabolism of sarcomas, specifically rhabdomyosarcoma (RMS) and osteosarcoma (OS), has not been characterized.

**Methods:**

Classical NF-κB activity was inhibited through overexpression of the IκBα super repressor of NF-κB in RMS and OS cells. Global gene expression analysis was performed using Affymetrix GeneChip Human Transcriptome Array 2.0, and data were interpreted using gene set enrichment analysis. Seahorse Bioscience XF^e^24 was used to analyze oxygen consumption rate as a measure of aerobic respiration.

**Results:**

Inhibition of classical NF-κB activity in sarcoma cell lines restored alternative signaling as well as an increased oxidative respiratory metabolic phenotype *in vitro*. In addition, microarray analysis indicated that inhibition of NF-κB in sarcoma cells reduced glycolysis. We showed that a glycolytic gene, hexokinase (HK) 2, is a direct NF-κB transcriptional target. Knockdown of HK2 shifted the metabolic profile in sarcoma cells away from aerobic glycolysis, and re-expression of HK2 rescued the metabolic shift induced by inhibition of NF-κB activity in OS cells.

**Conclusion:**

These findings suggest that classical signaling of NF-κB plays a crucial role in the metabolic profile of pediatric sarcomas potentially through the regulation of HK2.

## Introduction

Altered metabolism is considered a hallmark feature of cancer (1). Numerous studies have attempted to understand how cancer cells exploit this deregulation of cellular bioenergetics to their advantage. Normal differentiated cells use oxidative phosphorylation (OXPHOS) to generate ATP and metabolize glucose to pyruvate through glycolysis and then oxidize the pyruvate through the tricarboxylic acid (TCA) cycle to produce ATP (2). By contrast, cancer cells utilize a differential metabolic process, known as the “Warburg effect” or “aerobic glycolysis,” where the lower energy-producing glycolysis pathway is favored over the higher energy-yielding TCA and OXPHOS respiration pathways regardless of the availability of oxygen (3, 4). Cancer cells consume glucose at a higher rate and tend to convert most of their glucose into lactate (5). To sustain their growth and compensate for their higher metabolic demand, cancer cells drastically increase their amino acid uptake and the oxidative use of lipids, glutamine, and lactate (6–8). This altered metabolic reprogramming has been deemed important as it provides the cancer cells with essential nutrients necessary for their growth and proliferation such as nucleotides, lipids, NAD^+^, NADPH, and macromolecules (9, 10). Recent studies have shown that oncogenes like c-Myc, nuclear factor kappa B (NF-κB), tyrosine kinase receptors, and epidermal growth factors can turn on Ras/Raf or Akt-mediated pathways along with hypoxia-induced factor (HIF) to stimulate transcription of glycolytic genes to metabolically reprogram tumor cells (1–13).

Hexokinases (HKs) catalyze the phosphorylation of glucose (or fructose) to glucose-6-phosphate, the first irreversible step during glycolysis. HKs comprise of a family of four isoforms among which HK1 and HK2 are predominantly expressed. HK1 is generally ubiquitously expressed while HK2 expression is restricted to adipocytes and skeletal and cardiac muscle. Both HK1 and HK2 are known to associate with mitochondria and play an important role in cell survival (1, 4, 14). Numerous reports have indicated that HK2 is overexpressed in various cancers and is at least in part responsible for the increased glycolytic flux in tumor cells (1, 6, 15). It has been shown that the elevated expression of HK2 can be induced by both HIF1 and c-Myc (16, 17). HK2 is also believed to play a role in tumor initiation (18), and its high expression associates with oncogenic transformation and poor prognosis (1–23). Given its restricted distribution pattern in normal adult tissues and selective overexpression in cancer cells, HK2 has the potential to be a promising candidate for anticancer therapy (24).

Nuclear factor κB is a transcription factor that can exist as a homo or hetero dimer and is composed of five subunits, namely, RelA (known as p65), c-Rel, RelB, p50, and p52 (25). NF-κB is activated through two signaling pathways, the classical or the canonical pathway and the alternative or the non-canonical pathway (26). The regulation of classical NF-κB occurs through the upstream kinase inhibitor of κB kinase (IKK), consisting of two catalytic subunits IκB kinase α (IKKα) and IKKβ and a regulatory subunit IKKγ/NEMO. Phosphorylation of IKK leads to its activation and subsequent phosphorylation of the inhibitory protein IκB. This phosphorylation causes ubiquitination and subsequent degradation of IκB allowing NF-κB to translocate to the nucleus to activate target gene expression (27). Contrary to classical NF-κB signaling, alternative NF-κB is regulated by an IKKα homodimer complex, which phosphorylates the p100 precursor protein. This results in partial proteolysis of p100 and formation of the mature p52 subunit of NF-κB (28). The p52 subunit forms a heterodimer with RelB, which then translocates to the nucleus to bind NF-κB consensus binding sites and stimulate transcription, which is considered generally distinct from gene targets in the classical pathway (28).

Both the classical and the alternative NF-κB signaling pathways regulate many cellular processes including immune responses, proliferation, cell survival, apoptosis, lymphogenesis, and B cell maturation (29). In addition to these functions, both NF-κB pathways are involved in the regulation of skeletal muscle cell differentiation (30–32). In normal proliferating myoblasts, NF-κB maintains the expression of a repressor, YY1, which in turn inhibits the synthesis of the pro-myogenic microRNA, miR-29. When differentiation proceeds, NF-κB is downregulated, leading to a downregulation of YY1 and derepression of miR-29, allowing the expression of muscle-specific genes (32). By contrast, the alternative NF-κB pathway is activated late during myogenesis, and functions by promoting mitochondrial biogenesis to control oxidative metabolism in skeletal muscle (30). This occurs through direct transcriptional activation of mitochondrial regulator PPAR-γ coactivator 1β (PGC-1β), and more recently through the coordinated activity of the skeletal muscle master switch transcription factor, MyoD (33, 34).

In this study, we show that classical NF-κB signaling is in part responsible for causing a metabolic switch in sarcoma cells. Activation of classical NF-κB is associated with inhibition of the alternate pathway in these tumor types, and in addition promotes HK2 expression, which combined leads to a loss of oxidative metabolism in rhabdomyosarcoma (RMS) and osteosarcoma (OS) cells.

## Materials and Methods

### Cell Lines and Tumor Samples

Canine OS cell lines, OSA8, OSA16, OSA32, and OSA36, were provided by Dr. Jaime Modiano (University of Minnesota, Minneapolis, MN, USA) (35–37). The canine D17 OS cell line and human U2OS cell line were purchased from American Type Cell Culture Collection (ATCC, Manassas, VA, USA). All canine OS cell lines were maintained in RPMI-1640 (Gibco Life Technologies, Grand Island, NY, USA) supplemented with 10% fetal bovine serum, non-essential amino acids, sodium pyruvate, penicillin, streptomycin, l-glutamine, and HEPES (4-(2-dydroxethyl)-1-piperazineethanesulfonic acid) at 37°C, supplemented with 5% CO_2_ (media supplements from Gibco). Normal canine osteoblasts (catalog no. Cn406-05) (Cell Applications Inc., San Diego, CA, USA) were cultured in canine osteoblast medium (Cell Applications Inc., catalog no. Cn417-500). The human RMS RH30 cell line was established at St. Jude Children’s Research Hospital, and characteristics have been described (38). Unless otherwise mentioned, all the cell lines used were cultured in DMEM supplemented with 10% FBS. The human OS cell lines OS-1 and OS-17 and human Ewing sarcoma cell lines EW5, ES-1, ES-2, ES-7, and ES-8 were generously provided by the Pediatric Preclinical Testing Program. These lines were maintained by serial passaging in severe combined immune deficient mice as described previously by the Pediatric Preclinical Testing Program (39). Normal canine skeletal muscle tissue collections were approved by the OSU IACUC (protocol 2010A0015). Fresh frozen canine OS tumor samples were obtained from dogs presenting to The Ohio State University Veterinary Medical Center (OSU-VMC). Tumor sample collections were performed in accordance with established OSU-VMC hospital protocols and approved by the OSU IACUC (protocol 2010A0015).

### Transfections and Infections

For luciferase assays, cells were transfected in 12-well plates, and luciferase activity was monitored as previously described (40). pBabe-puro was used for expressing IκBα SR. pCL-Eco was used for expressing IKKα ΔNBD. pSIREN-RetroQ was used for expressing shHK2. pWZL-Neo-Myr-Flag-HK2 was obtained from Addgene (Cambridge, MA, USA) (Plasmid 20501) (41). Retroviruses were prepared by calcium phosphate-mediated gene transfection kit (Promega, Madison, WI, USA) as previously described (42). RH30 and U20S cells were infected by applying 4–5 cfu/cell viruses in a total volume of 4 mL full culture medium containing 4 µg/mL polybrene for 8 h. Cells were transfected with 10 nM of AllStars negative control siRNA or p65-targeting siRNA (5′-CTATGATGAGTTTCCCACCATGGTG-3′) according to standard protocols using Lipofectamine 2000.

### Western Blot Analysis

Cell extracts were made by lysing PBS-washed cell pellets in radio-immunoprecipitation assay buffer supplemented with complete Protease Inhibitor Cocktail (Roche Diagnostics, Indianapolis, IN, USA). Canine cell extracts were made by resuspending the cells in lysis buffer consisting of 20 mM Tris–HCl pH 8.0, 137 mM NaCl, 10% glycerol, 1% IPEGAL CA-630, 10 mM ethylenediaminetetraacetic acid, 1 mg mL^−1^ aprotinin, 1 mg mL^−1^ leupeptin, 1 mg mL^−1^ pepstatin A, 1 mM phenylmethylsulphonyl fluoride, 1 mM sodium orthovanadate, and 10 mM sodium fluoride (all from Sigma, St. Louis, MO, USA) for 1 h at 4°C. In addition, protein lysates were prepared from frozen canine normal muscle or OS tumor samples by pulverizing tissues in liquid nitrogen and resuspending them in the same lysis buffer with proteinase and phosphatase inhibitors. For each sample, 30 µg of protein was loaded on each gel. Proteins were transferred onto a PVDF membrane using a tank blotter (Bio-Rad, Hercules, CA, USA). The membranes were then blocked using 5% milk and 1× Tris-buffered saline plus Tween 20 (TBST) and incubated with primary antibody overnight at 4°C. Membranes were then washed with 1× TBST and incubated with the corresponding secondary antibody. Membranes were again washed with 1× TBST, incubated with SuperSignal West Pico Chemiluminescent Substrate according to the manufacturer’s protocol (Thermo Fisher Scientific, Waltham, MA, USA), and visualized by autoradiography. The antibodies used included anti-p65 (Millipore, Billerica, MA, USA; Cell Signaling, Danvers, MA, USA), anti-p52 (Cell Signaling), anti-p100 (Cell Signaling), anti-IκBα (Santa Cruz Biotechnology, Dallas, TX, USA), and anti-HK2 (Cell Signaling). Anti-GAPDH (Santa Cruz Biotechnology) and anti-α-Tubulin (Sigma) were used as loading controls.

### Electrophoretic Mobility Shift Assay (EMSA) and Gene Expression Analysis

Electrophoretic mobility shift assays were performed as previously described (40). For quantitative RNA expression, total RNA was isolated from cells by Trizol extractions (Invitrogen). Two micrograms of total RNA were reversed transcribed with MultiScribe™ MuLV reverse transcriptase (Applied Biosystems, Foster City, CA, USA). cDNA equivalent to 40 ng was used for quantitative polymerase chain reaction (qPCR) amplification (Applied Biosystems) with SYBR green PCR master mix (Applied Biosystems). Samples in which no reverse transcriptase was added (no RT) were included for each RNA sample. The relative levels of expression of genes were normalized according to those of human GAPDH (5′-GGGGAGCCAAAAGGGTCATC-3′, 5′-GGCATGGACTGTGGTCATGAG-3′). qPCR data were calculated using the comparative Ct method (Applied Biosystems). Primers to the coding region of human HK2, 5′-GAATGGGAAGTGGGGTGGAG-3′ and 5′-TCTCGTCCAGGCTGTTCTGC-3′ were used for qRT-PCR analysis. Where possible, intron spanning primers were used. All qPCR reactions were performed in triplicate, and three independent RNA samples were assayed for each time point.

### Chromatin Immunoprecipitation (ChIP) Assay

Chromatin immunoprecipitation assays were performed and quantified as described previously (43, 44) with the following modifications: 1 × 10^7^ cells were used for each immunoprecipitation, and protein A agarose beads (Invitrogen) were used to immunoprecipitate the antibody:antigen complexes. The assays were performed using an antibody against p65 (Millipore). Rabbit IgG (Millipore) was used as a non-specific control. Primers spanning the NF-κB binding sites on the *HK2* (site 1 TCTGGCCTCCATCAGTCTCT, site 2 CCCTCCAGTAACAGGGAAAA, site 2 GTGTAGGAGACGAGCGGTTC, site 2 AGAGGCAGATCCTGAGGACA, site 3 ATGGGGGTCATCTCTCTTCC, site 3 GCACACTGCAGCGCTTAAT) gene and IgH (GCCGATCAGAACCAGAACACCTGC and TGGTGGGGCTGGACAGAGTGTTTC) were used to detect enrichment. Real-time PCR reactions were performed in triplicate. Values of [Δ][Δ]Ct were calculated using the following formula based on the comparative Ct method: [Δ]Ct, template (antibody) − [Δ]Ct, template (IgG) = [Δ][Δ]Ct. Fold enrichments were determined using the formula: 2^−[Δ][Δ]Ct^ (experimental)/2^−[Δ][Δ]Ct^ (reference, IgH). SE from the mean was calculated from replicate [Δ][Δ]Ct values. The IgH locus was used to normalize the fold enrichments for the individual promoters. All ChIP assays shown are representative of at least three individual experiments.

### Seahorse Analysis

The metabolic profile of RMS and OS cells was determined using Seahorse XF^e^24 Extracellular Flux Bioanalyzer (Agilent) to measure basal and maximal oxygen consumption rate (OCR), an indicator of mitochondrial respiration, as suggested by the manufacturer. The Seahorse Bioanalyzer provides real-time measurements of OCR as a measure of OXPHOS. In brief, cells were incubated for 1 h in DMEM supplemented with 10 mM glucose, 2 mM l-glutamine, and 1 mM sodium pyruvate. For the MitoStress test, cells were analyzed under basal conditions and after injection of three inhibitors: 1 µM oligomycin, 0.3 µM carbonyl cyanide 4-trifluoromethoxy-phenylhydrazone (FCCP), and 0.5 µM rotenone. Oligomycin inhibits ATP synthase, which allows measurement of ATP-coupled oxygen consumption through OXPHOS. FCCP is an uncoupling agent that allows maximum electron transport, therefore a measure of maximum OXPHOS capacity. Rotenone is an inhibitor of complex I of the mitochondrial respiratory chain that allows calculation of the rate of respiration solely attributable to mitochondria. ATP-linked respiration was calculated by subtracting the lowest OCR value after administrating of oligomycin from the third basal OCR measurement. All chemicals were obtained from Sigma.

### Proliferation and Cell Death Analyses

For growth analysis, cells were plated in triplicate into 24-well plates, and the cells were trypsinized and counted each day. Cell growth was also determined using time-lapse live cell imaging. Cells were seeded in triplicate in 96-well plates at a density of 3,000 cells/well and placed in the IncuCyte Zoom (Essen Biosciences, Ann Arbor, MI, USA) in 37°C and 5% CO_2_ conditions. Three high-definition phase-contrast images were collected from each well every hour. IncuCyte Zoom 2015A software (Essen Bioscience) was used for analysis to apply a mask that determined the percent confluence based on the phase-contrast images. The percent confluence was normalized to the initial seeding confluence. For cell death assays, cells were seeded in 96-well plates as described earlier, and Caspase-Glo 3/7 assay (Promega) was performed according to the manufacturer’s protocol. Annexin V (BD Biosciences, Franklin Lakes, NJ, USA) and propidium iodide (Sigma) were used to stain cells, and flow cytometry was used for determining the percentage of apoptotic cells.

### Gene Expression Microarray and Gene Set Enrichment Analysis (GSEA)

Total RNA was isolated using a standard Trizol extraction protocol and purified with a cleanup kit (Qiagen). Affymetrix GeneChip Human Transcriptome Array 2.0 (Affymetrix) arrays were used for microarray. Probe intensity (CEL) files were obtained, and Affymetrix Transcriptome Analysis Console software was used for array analysis. Differentially expressed genes were identified by the Robust Multichip Average method (45).

Gene set enrichment analysis v2.0 was used to determine the enrichment of functional categories in the entire linear gene expression dataset (46). GSEA was performed using MSigDB C2 CP: Canonical pathways gene set collection. Statistical significance was determined using 1,000 random permutations of each gene set to obtain a nominal *p*-value and false discovery rate. Heat map was generated using GenePattern (47). HeatmapViewer (v11) was used to display values in heat map format for the genes identified from GSEA analysis.

The microarray data have been deposited into the GEO database with the accession number GSE112119.

### Statistics

Quantitative data are presented as mean ± SEM. Statistical analysis was performed using GraphPad Prism software. Statistical comparisons were performed using unpaired two-tailed Student’s *t* tests, with a probability value of <0.05 taken to indicate significance.

## Results

### Sarcomagenesis Associates With a Shift in NF-κB Activity From the Alternative to the Classical Pathway

**Figure 1:**
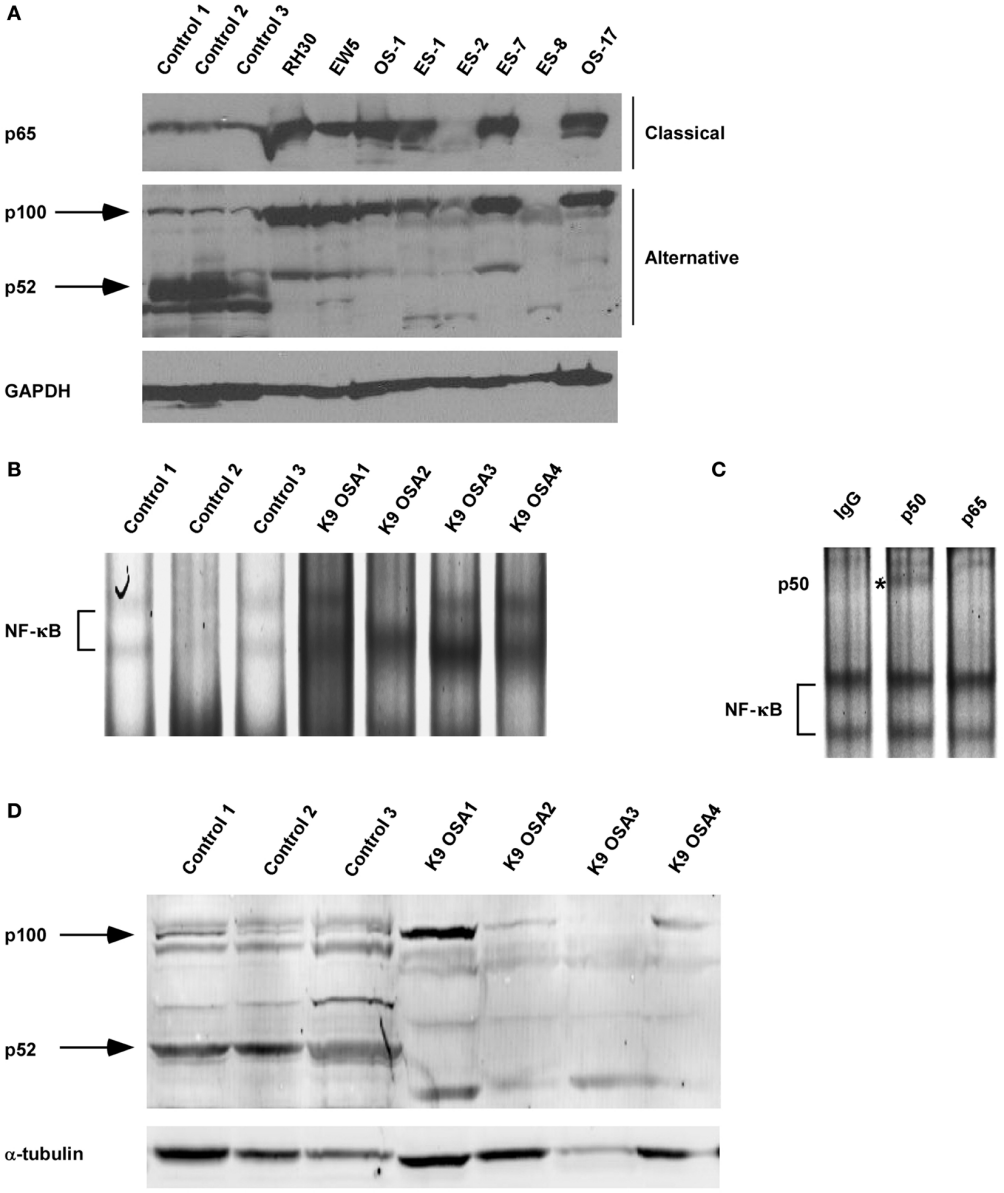
Nuclear factor κB (NF-κB) activity shifts from the alternative to the classical pathway during sarcomagenesis. **(A)** Xenografted tumors from eight different sarcoma cell lines were isolated. RH30 is a rhabdomyosarcoma cell line. OS-1 and OS-17 are osteosarcoma (OS) cell lines. EW5, ES-1, ES02, ES-7, and ES-8 are Ewing sarcoma cell lines. Control tissue protein extracts were isolated from normal muscle tissues. All tissues were isolated from four independent mice and homogenized to make protein extracts. Nuclear extract was isolated for p65 western blot. Western blots were performed by loading equal amounts of protein using antibodies against subunits of the classical and alternative NF-κB pathways. GAPDH was used as a loading control. **(B)** Electrophoretic mobility shift assays were performed on nuclear protein extracts isolated from primary canine OS tissues and adjacent controls tissue. **(C)** Supershift analysis was performed on the same nuclear extract isolated for sample K9 OSA3 in panel **(B)**. The antibodies used were raised against rabbit IgG, p50, or p65. ns denotes non-specific band. **(D)** Protein extracts from primary canine OS tissues and adjacent control tissue were made, and western blots were performed using antibodies as described in panel **(A)**.

Our previous research using the murine C2C12 myoblast cell line showed that alternative NF-κB signaling is activated during myogenesis to promote OXPHOS in myotubes (3, 48). Interestingly, RMS tumors are thought to derive from a skeletal muscle lineage that improperly differentiates and exhibits defects in mitochondrial gene expression, favoring a Warburg effect (49, 50). Given these observations, we asked whether NF-κB signaling pathways were altered in RMS. We first performed western blot analysis on tissue extracts from xenografted human RH30 RMS tumors compared with normal skeletal muscle tissue. While normal tissue exhibited low levels of nuclear p65, processing of p100 to p52 was readily visible, indicative of an activated alternative signaling pathway (Figure 1A; Figure S1 in Supplementary Material). By contrast, in RH30 tumors, nuclear p65 expression was markedly increased while p100 processing to p52 was impaired (Figure 1A). In addition to human samples, we examined NF-κB signaling pathways in canine OS tumors. Attempts to use various p65 commercially available antibodies were unsuccessful in recognizing the canine form of the NF-κB subunit. Hence, we probed for NF-κB by EMSA using both canine control and OS samples. Results showed increased DNA binding activity in tumor samples, which by supershift analysis contained the p50 subunit of NF-κB (Figures 1B,C). This is evidence that classical signaling is increased in canine OS tumors. Similar to human sarcomas, we also observed that alternative signaling was decreased in canine OS tumors (Figure 1D). Together, results suggest that during the development of sarcomas, an NF-κB signaling switch occurs that causes the inactivation of the alternative pathway and the activation of the NF-κB classical pathway.

**Figure 2:**
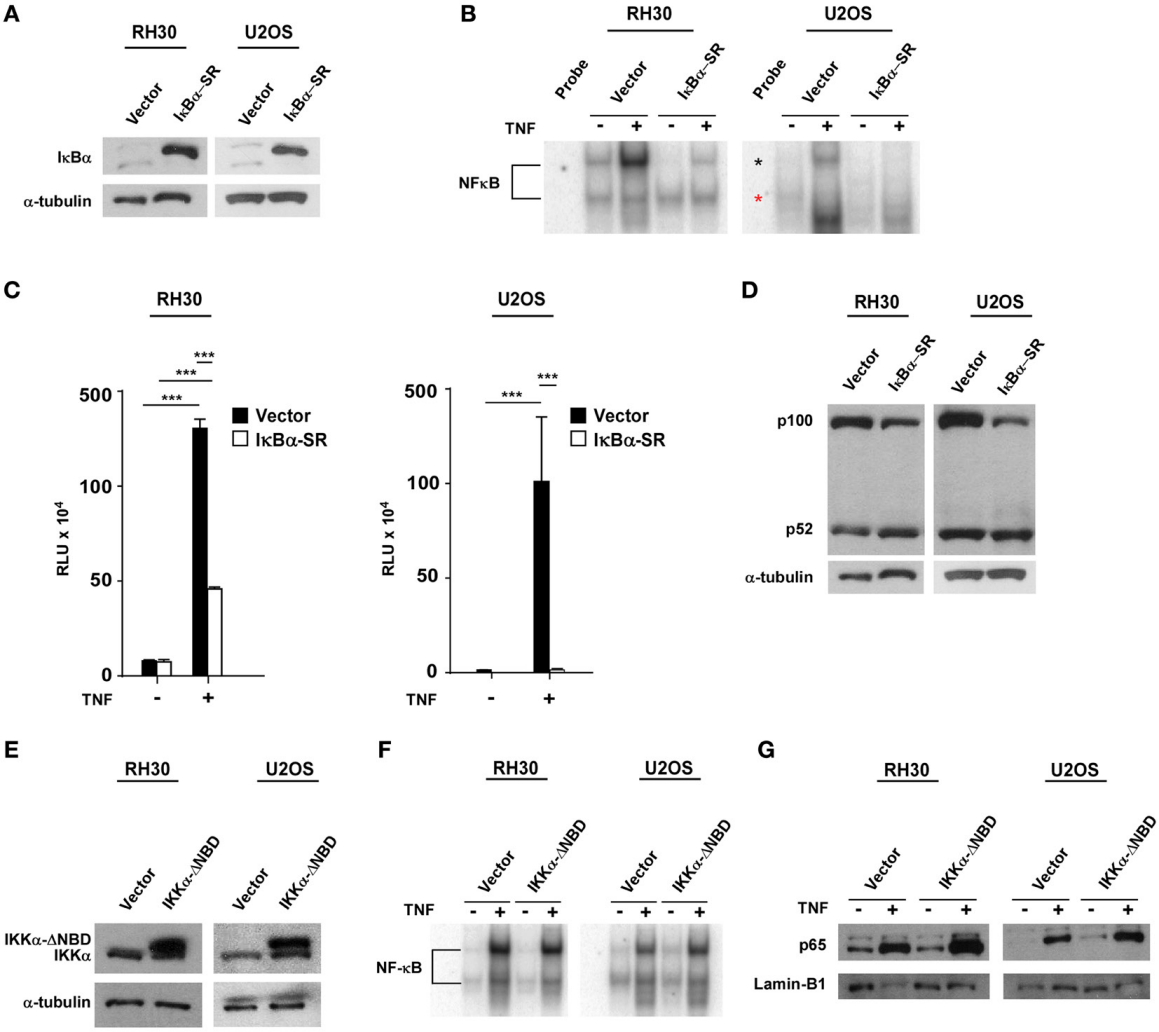
Establishment of RH30/U2OS-IκBα SR and relationship between classical and alternative nuclear factor κB (NF-κB) pathways. **(A)** Stable cell lines overexpressing the transdominant inhibitor IκBα transgene were established in both RH30 and U20S cells. Western blots were performed with an IκBα antibody to confirm overexpression of IκBα. RH30 and U20S vector cells were used as controls. Tubulin was used as a loading control. **(B)** RH30/U2OS vector and RH30/U2OS-IκBα SR cells were treated with 5 ng/mL tumor necrosis factor (TNF) α. Nuclear proteins were extracted and incubated with ^32^P-labeled NF-κB binding probe. Samples were separated on 5% acrylamide gel, and electrophoretic mobility shift assays (EMSAs) were performed. **(C)** The vector containing luciferase gene under regulation of 3xκB and cmv-LacZ vector were co-transfected into RH30/U2OS-vector and RH30/U2OS-IκBα SR cell lines. Luciferase activity was measured under basal and TNFα-stimulated conditions. Transfection efficiency was normalized by β-galactosidase activity. Data represent average ± SEM from three independent experiments. **(D)** Protein extracts were made from both RH30 and U20S vector and IκBα cells. Western blots were performed using antibodies against p52 and p100, the subunits of the alternate NF-κB signaling pathway. Tubulin was used as a loading control. **(E)** Western blots were probed with an IκB kinase α (IKKα) antibody in vector and IKKα ΔNBD-expressing RH30 and U2OS cells. **(F)** EMSA was performed with vector and IKKα ΔNBD-expressing RH30 and U2OS cells in the absence or presence of TNF. **(G)** Collected nuclear extracts were analyzed by western blot to detect activation status of the classical NF-κB pathway in vector and IKKα ΔNBD-expressing RH30 and U2OS cells in the absence or presence of TNF. Cells were treated with TNF (***p < 0.01 relative to control).

Next, we asked whether NF-κB signaling pathways were coupled during sarcomagenesis. To examine this regulation, we established RH30 and U2OS cell lines stably expressing the IκBα super repressor inhibitor of NF-κB (RH30/U2OS-IκBα SR) (Figure 2A). EMSAs and NF-κB cell luciferase reporter assays were used to confirm the efficiency of IκBα SR on the inhibition of NF-κB activity in RH30 and U2OS cells treated with the NF-κB activating cytokine, tumor necrosis factor (TNF) (Figures 2B,C). We then analyzed whether the inhibition of classical NF-κB signals impacted the alternative pathway in sarcoma cells. Compared with the p100 to p52 processing observed in RH30/U2OS-vector cells, there was a modest, but noticeable increase in p100 to p52 processing in RH30/U2OS-IκBα SR cells (Figure 2D). This suggested that the activation of the classical pathway occurs in the context of diminished alternative signaling, potentially driving the shift in NF-κB pathways observed in sarcomas.

To determine whether the loss of alternative signaling in sarcomas was reversibly coupled to the classical pathway, we generated RH30 and U2OS cells stably expressing an inactive form of the IKKα subunit where the NEMO-binding domain (NBD) had been deleted (IKKα ΔNBD) (Figure 2E). This domain is normally required for IKKα to form a heterotrimeric complex with IKKβ and IKKγ (NEMO) to activate classical NF-κB signaling. Deletion of NBD predominantly forces IKKα dimerization and activation of the alternative pathway (51). When we examined stable RH30 and U2OS IKKα ΔNBD clones for effects on classical pathway activation, no difference in p65 DNA binding activity was detected by EMSA compared with vector control cells (Figure 2F). Similarly, no difference was observed by western blot when probing for the nuclear localization of p65 in RH30 and U2OS IKKα ΔNBD clones versus controls (Figure 2G). These findings suggested that activation of alternative signaling does not affect the reciprocal activation of the classical pathway. Thus, the shift in NF-κB pathway activity in sarcoma cells likely results from a dominant classical signaling pathway, which when activated causes a concomitant reduction in alternative pathway activity.

### Classical NF-κB Inhibits Mitochondrial Oxidative Metabolism in Sarcoma Cells

**Figure 3:**
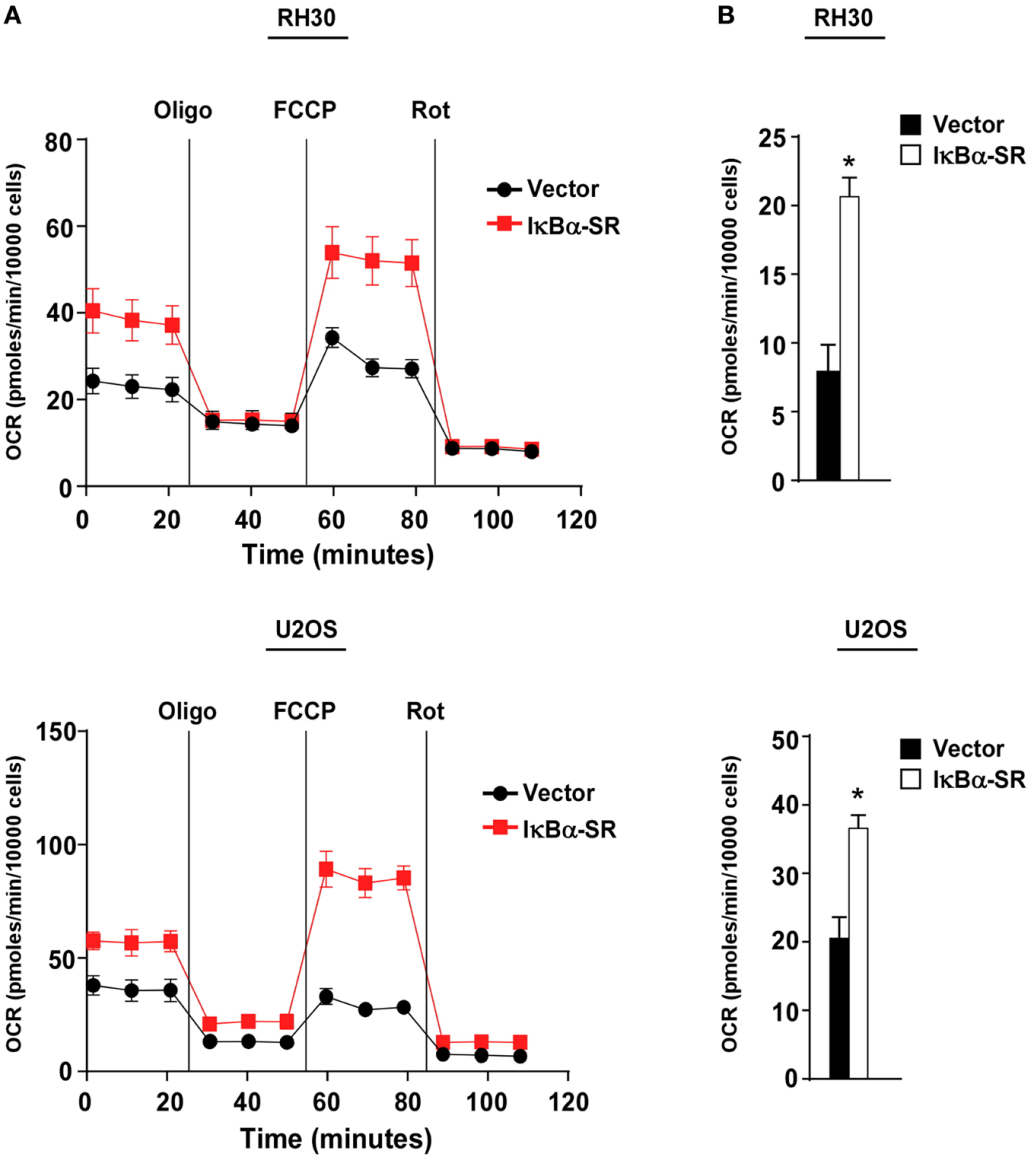
Classical nuclear factor κB pathway inhibits mitochondrial oxidative metabolism in sarcoma cells. **(A)** Oxygen consumption rate was measured in RH30 and U2OS vector and RH30/U2OS-IκBα SR cells under basal conditions and in the presence of oligomycin, FCCP, and rotenone. **(B)** ATP-linked respiration calculated based on Figure 3A. Data represent average ± SEM from three independent experiments (*p < 0.05 relative to control).

We previously demonstrated that alternative signaling of NF-κB is important in regulating oxidative metabolism in skeletal muscle cells (30, 33, 34). Since this same pathway is downregulated in sarcoma cells, we asked whether the forced inactivation of classical NF-κB signaling in sarcoma cells, which we showed in Figure 2 partially re-establishes alternative signaling, would also promote oxidative metabolism. To examine the bioenergetic profile of sarcoma cells, we used the Seahorse Bioscience XF^e^24 extracellular flux analyzer to measure OCR with a Mito stress test. OCR was used as a measure of aerobic respiration comparing RH30/U2OS vector and RH30/U2OS-IκBα cells. RH30/U2OS-IκBα SR cells lacking NF-κB activity had a substantially increased OCR profile compared with vector control cells (Figure 3A). Treatment of these cells with the ATP synthase inhibitor oligomycin, which blocks a necessary step in OXPHOS for ATP production, also revealed a significant increase (*p* < 0.05) in ATP-linked respiration (Figure 3B). Knockdown of p65 using siRNA recapitulated the metabolic effect seen with IκBα SR expression in U2OS cells (*p* < 0.05), but not in RH30 cells (Figure S2A in Supplementary Material). When we activated the alternative pathway by expressing IKKα ΔNBD, no metabolic difference was observed in RH30 or U2OS cells (Figure S2B in Supplementary Material), reaffirming our earlier results that alternative signaling does not reverse the activation of the classical pathway. Thus, findings suggest that activation of classical NF-κB reduces mitochondrial oxidative metabolism in sarcoma cells.

### NF-κB Is a Direct Transcriptional Regulator of HK2

**Figure 4:**
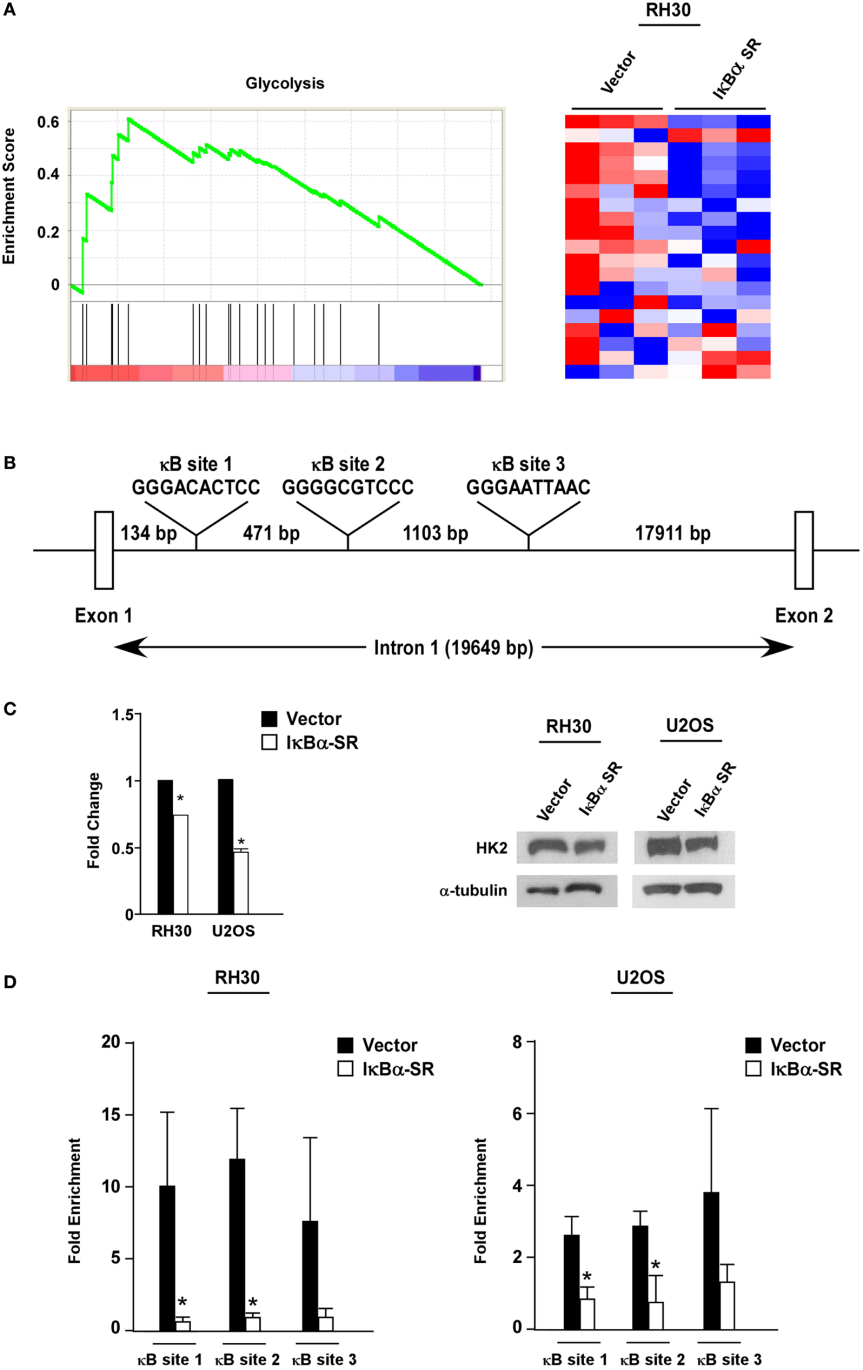
Nuclear factor κB (NF-κB) regulates hexokinase (HK) 2 in sarcoma cells. **(A)** Gene set enrichment analysis plot of MSigDB Mootha_Glycolysis gene signature in vector and IκBα SR expressing RH30 cells. Gene expression profile data were obtained by Affymetrix GeneChip Human Transcriptome Array 2.0. Nominal *p*-value < 0.05, FDR < 0.25. Heat map showing differentially expressed glycolysis genes in vector and IκBα SR expressing cells. **(B)** Schematic representation of the HK2 gene indicating the locations of the conserved NF-κB consensus binding sites. **(C)** RNA was reverse transcribed, and real-time quantitative PCR analysis was performed with primers against the HK2 gene. Primers to GAPDH were used to normalize the samples. Data are shown as fold stimulation with each sample compared relative to the untreated sample. Error bars represent SD from the mean for the replicate values. Western blots were performed using protein extracts prepared from RH30 and U20S vector and IκBα SR cells. Tubulin was used as a loading control. Data represent average ± SEM from three independent experiments. The western blot is a representative of three independent experiments. **(D)** Chromatin immunoprecipitation assays were performed in RH30 and U20S vector and SR cells using antibodies against p65. DNA was precipitated, and real-time PCR was performed with primers specific to sites 1–3 on the HK2 gene. IgG was used as a control, and the relative enrichment at the IgH locus was used to normalize the data. Three biological replicates are represented in the graphs, and error bars represent SD. Chromatin immunoprecipitation assay was done with anti-p65 and IgG antibodies. Recovered DNA fragments were subjected to real-time PCR analysis with primer sets surrounding κB sites 1–3. Values were represented as enrichments relative to inputs (*p < 0.05 relative to control).

To probe the potential mechanism by which NF-κB regulates metabolism in sarcoma cells, we performed global gene expression analysis comparing RMS cells lacking NF-κB activity versus vector control cells. Using GSEA, we found that a gene set for glycolysis was significantly enriched (nominal *p* < 0.05; FDR < 0.25) in the control cells compared with the cells lacking NF-κB activity (Figure 4A). Since glycolytic genes showed decreased expression with loss of NF-κB activity, we examined these genes by rVista for potential conserved NF-κB binding sites. Results revealed HK2 as a potential transcriptional target of NF-κB, containing multiple binding sites in the first intron (Figure 4B). Consistent with the notion that HK2 is regulated by NF-κB, we observed that HK2 expression was reduced in RH30/U2OS-IκBα SR compared with vector control cells (Figure 4C). To further examine whether HK2 is regulated by NF-κB, we performed knockdown of p65 and observed decreased expression of HK2 in RH30 and U2OS cells silenced for p65 (Figure S3 in Supplementary Material). This suggested that NF-κB is a regulator of HK2 in sarcoma cells.

To determine whether HK2 is a direct target gene of NF-κB, we next performed ChIP assays using primers against three conserved NF-κB binding sites within the first intron of the HK2 gene located at positions +2123, +2604 and +3717 (named sites 1, 2, and 3, respectively) (Figure 4B). Results showed that p65 was significantly bound to all three NF-κB binding sites in both RH30 and U20S cells, and importantly, that this binding was reduced in sarcoma cells expressing the IκBα SR transgene (Figure 4D). Such data are supportive that classical NF-κB is a direct transcriptional regulator of HK2.

### HK2 Is Required for the Metabolic Phenotype, but Not Proliferation, of Sarcoma Cells

**Figure 5:**
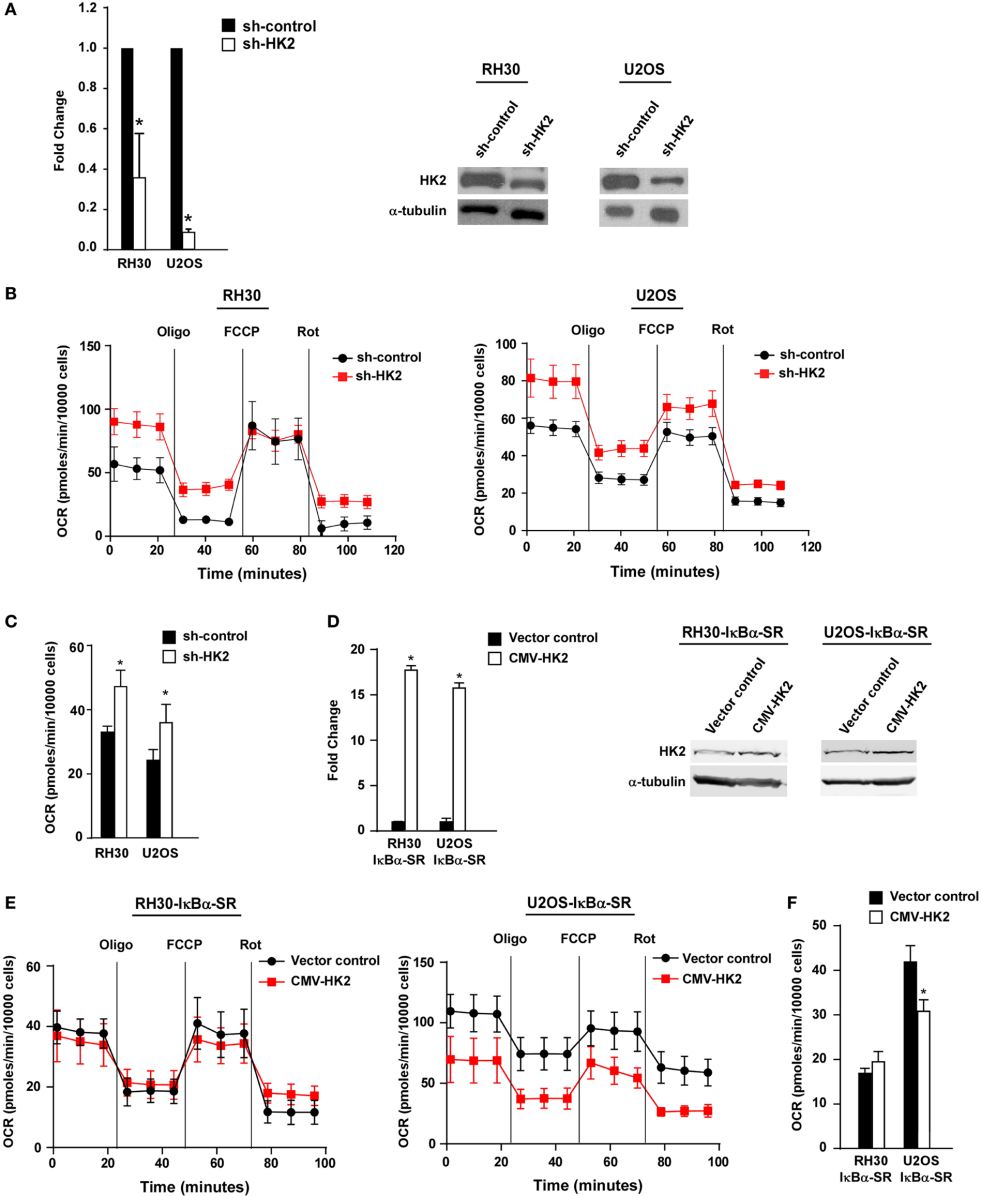
Knockdown of hexokinase (HK) 2 restores oxidative metabolism in sarcoma cells. **(A)** HK2 was stably knocked down using short hairpin RNA (shRNA) constructs. Real-time PCR was performed on cDNA samples from two independent shRNA clones with primers against HK2. Knockdown of HK2 was also confirmed at the protein level by western blot using antibodies against HK2. Tubulin was used as a loading control. Data shown are average values ± SEM obtained from triplicates. **(B)** Oxygen consumption rate (OCR) was measured in RH30/U2OS vector and RH30/U2OS-shHK2 cells as described in Figure 3A. Data represent average ± SEM from three independent experiments. **(C)** ATP-linked respiration was measured in RH30/U2OS-SR vector and RH30/U2OS-SR-HK2 cells as described in Figure 3B. **(D)** HK2 was overexpressed in RH30/U2OS-IκBα SR cells. Real-time PCR was performed on cDNA samples with primers against HK2. **(E)** OCR was measured in RH30/U2OS-IκBα SR cells with enforced expression of HK2 compared with vector control as described in Figure 3A. **(F)** ATP-linked respiration was calculated from panel **(E)** (*p < 0.05 relative to control).

Previous reports have shown that HK2 is an important regulator of tumor growth and the Warburg effect (52–54). To determine if a similar role existed in sarcoma cells, we silenced HK2 in RH30 and U2OS cells using a short hairpin RNA (shRNA) that was confirmed by qRT-PCR and western blotting analysis (Figure 5A). Proliferation assays showed that the growth rate of RH30/U2OS-HK2 shRNA cells were not significantly different compared with RH30/U2OS-control shRNA cells (Figures S4A,B in Supplementary Material), nor did we observe an increase in cell death in RH30/U2OS-HK2 shRNA cells compared with RH30/U2OS-control shRNA cells (Figures S4C,D in Supplementary Material). These data indicated that HK2 was not required for sarcoma tumor cell proliferation or survival.

We next utilized the Seahorse Bioscience XF^e^24 extracellular flux analyzer to examine whether HK2 depletion affected the metabolic profile of sarcoma cells. Similar to what we observed in sarcoma cells devoid of NF-κB, RH30 and U2OS cells silenced for HK2 also exhibited an increase in ATP-linked mitochondrial oxygen consumption, suggestive of a shift away from the Warburg effect (Figures 5B,C). Results further showed that cells lacking HK2 expression displayed a higher oxidative metabolism. To determine whether HK2 regulation of metabolism in sarcoma cells was dependent on NF-κB, we induced HK2 expression in RH30 and U2OS IκBα SR cells (Figure 5D). Results showed that expression of HK2 rescued the metabolic shift seen in U2OS IκBα SR cells (*p* < 0.05), but not in RH30 cells (Figures 5E,F). Taken together, these data support the notion that HK2 function in sarcoma cells is dispensable for the proliferation and viability of these cells, but important as a regulator of metabolism.

## Discussion

Reprogramming of glucose metabolism called aerobic glycolysis or the Warburg effect is recognized as an important feature distinguishing cancer cells from normal cells (1). It is already well known that many cancer cells depend on glycolysis not only for ATP production but also for the synthesis of biomolecular precursor substrates, necessary for their rapid replication (55). This reprogramming is often related to enzymatic activation or transcriptional upregulation of glycolytic genes, which is caused by constitutive activation of oncogenic signaling pathways and inactivation of tumor suppressor genes (56). In this report, we show evidence suggesting that classical NF-κB promotes the Warburg effect in pediatric sarcomas, and identified HK2 as one of the glycolytic genes directly regulated by classical NF-κB. Our findings further suggest that a switch in NF-κB signaling from the alternative to the classical pathway occurs during sarcomagenesis that functionally promotes a metabolic reprogramming.

A significant feature of cancer development involves a shift in metabolism by increasing aerobic glycolysis while reducing OXPHOS (1). While the mechanisms underlying the Warburg effect continue to be elucidated, previous studies indicated that NF-κB influences metabolism through cross talk with transcription factors such as p53 and HIF (57, 58). Enhanced activation of NF-κB has also been shown to increase glucose uptake and glycolytic flux in *p53^−/−^* mouse embryonic fibroblasts through the upregulation of glucose transporter 3 (GLUT3) (59). In lymphoma, NF-κB was reported to promote the translocation of GLUT1 to the plasma membrane (60). Another study showed evidence that in the absence of p53, p65 can repress expression of mitochondrial genes and subsequently suppress OXPHOS (57). Previously in our lab, we also demonstrated that the alternative pathway replaced classical NF-κB during myogenesis, and activated alternative NF-κB promoted mitochondrial biogenesis, resulting in increased ATP production by OXPHOS (34). In comparison, others have showed that the classical NF-κB pathway promoted the Warburg effect by upregulating glycolytic genes, GLUT3 (59) and pyruvate kinase M2 (PKM2) (61), in cancer cells.

Based on these findings, we speculated that classical NF-κB might also regulate a metabolic profile of sarcoma cells. We first detected components of classical and alternative NF-κB pathways and showed that alternative NF-κB signaling is activated in normal tissues, which is replaced by classical signaling in sarcomas. We also found that both signaling pathways are coupled, in that the increase in classical signaling leads to the reduction in the alternative pathway during sarcomagenesis. Our findings further support that this shift in NF-κB signaling causes an inhibition of mitochondrial oxidative metabolism, which may account for the Warburg effect in RMS and OS tumors. Our results showed that inhibition of NF-κB by expression of the IκBα-SR transgene or an siRNA against p65 had a stronger effect on the metabolism of U2OS cells versus RH30. One possible explanation for this difference could be that in RH30 cells, other classical subunits of NF-κB compensate for p65. Therefore, expression of the IκBα-SR transgene would be expected to inhibit these other subunits to cause a shift in oxidative metabolism, as we observed, whereas simply inhibiting p65 would not have this same metabolic effect.

The exact nature of the mechanisms acting downstream of NF-κB in sarcoma metabolism warrants further investigation. Our current evidence points to HK2 as at least one key glycolytic enzyme involving NF-κB. HK2 catalyzes the first step of the glycolysis pathway and its reaction converting glucose to glucose-6-phosphate is essentially irreversible (24). Recent studies have identified that HK2 has functions in processes other than metabolism such as apoptosis (62), cell cycle (23), and autophagy (63). Considering the reports that PKM2 is also a protein kinase and transcriptional co-regulator, the importance of glycolytic genes for cancer cells has been growing with respect to not only metabolism but also the other hallmarks of cancer (64). Several clinical trials have attempted to improve patient outcome by inhibiting HK2 using a glucose analog, 2-deoxyglucose (2-DG). Although 2-DG is used as a stand-alone therapy, evidence indicates that 2-DG in combination with chemotherapy or radiotherapy is efficacious as an anticancer treatment (65, 66). In sarcoma cells, we found that HK2 was not required for cell proliferation or survival, but instead functioned as a regulator of metabolism. Results also revealed that HK2 is regulated by NF-κB in OS and RMS cells. However, overexpression of HK2 did not restore the metabolic profile in RH30 cells expressing the IκBα-SR transgene, suggesting that differences exist in the manner in which NF-κB regulates metabolism in RMS cells *via* HK2. We suspect that in RH30 cells, NF-κB is inhibiting oxidative metabolism by regulating multiple glycolytic factors including HK2, and simply re-expressing HK2 in cells that completely lack NF-κB activity is not sufficient to rescue the metabolic effect that is being maintained by other NF-κB regulated genes. Collectively, we propose that HK2 represents one of several glycolytic genes regulated by NF-κB that function in the metabolic reprogramming of sarcoma tumors. However, our finding that classical NF-κB is activated in RMS, OS, and Ewing sarcoma cells also leads us to believe that NF-κB signaling might be relevant in promoting a dysregulated metabolism in potentially multiple sarcoma subtypes.

## Author Contributions

The study was conceived by DG. PL, PY, and YI designed and performed experiments. PL, PY, YI, and DG wrote the manuscript. KL and JF helped perform experiments. PY performed bioinformatics analyses. CL and PH supported through interpretation of data for the work. All the authors revised the manuscript critically, approved the final manuscript, and agreed to be accountable for all aspects of the manuscript.

## Conflict of Interest Statement

The authors declare that the research was conducted in the absence of any commercial or financial relationships that could be construed as a potential conflict of interest.

## Abbreviations

2-DG, 2-deoxyglucose; EMSA, electrophoretic mobility shift assay; GLUT3, glucose transporter 3; HK, hexokinase; HIF, hypoxia-induced factor; IKK, inhibitor of κB kinase; NBD, NEMO-binding domain; NF-κB, nuclear factor κB; OCR, oxygen consumption rate; OXPHOS, oxidative phosphorylation; OS, osteosarcoma; PKM2, pyruvate kinase M2; RMS, rhabdomyosarcoma; TCA, tricarboxylic acid; TNF, tumor necrosis factor.
