# Supplementary figures and images for: Classical NF-κB Metabolically Reprograms Sarcoma Cells Through Regulation of Hexokinase 2
Source: Front Oncol. 2018 Apr 11;8:104. doi: 10.3389/fonc.2018.00104 (PMC5904193; doi:10.3389/fonc.2018.00104)

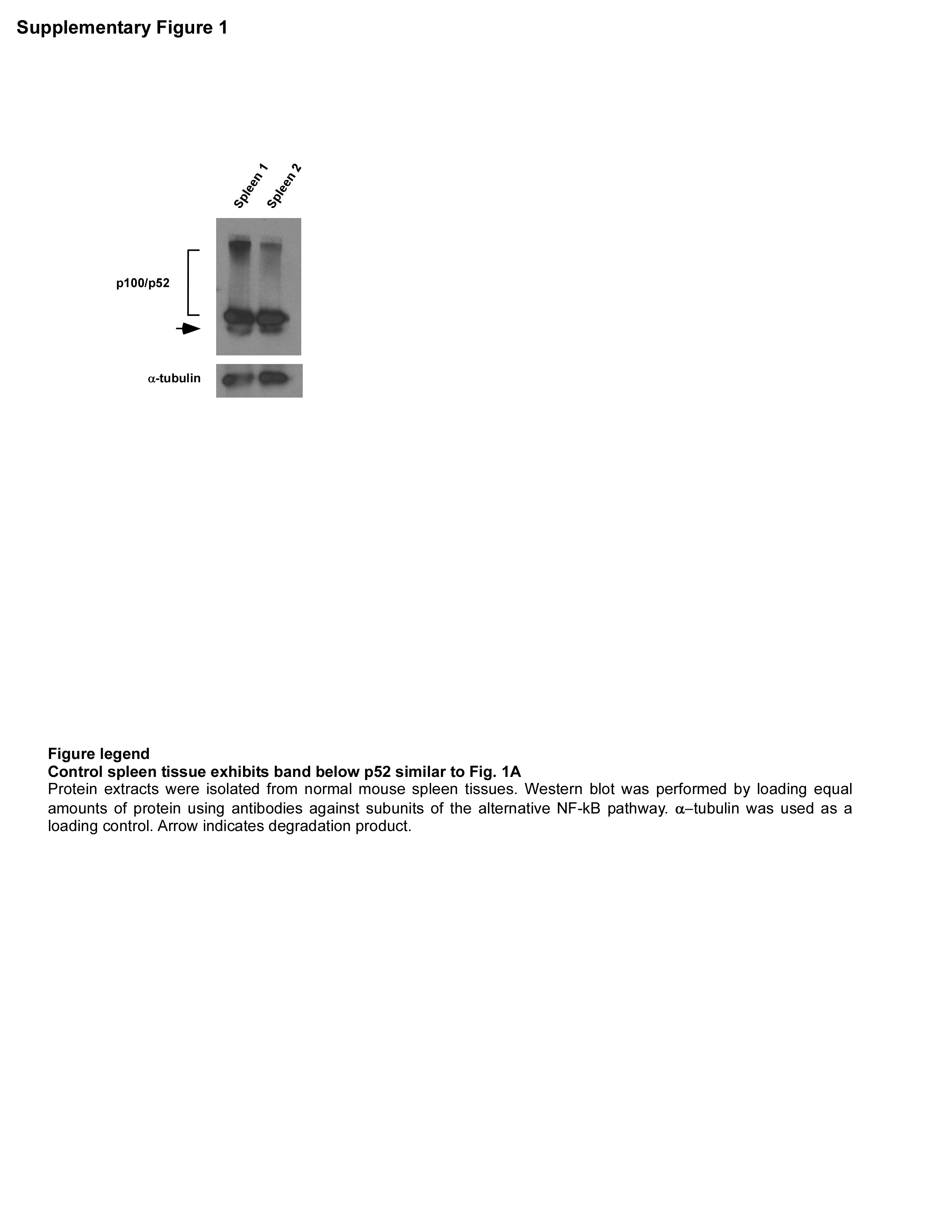

Supplement: Supplementary file 1 [file Image_1.JPEG]

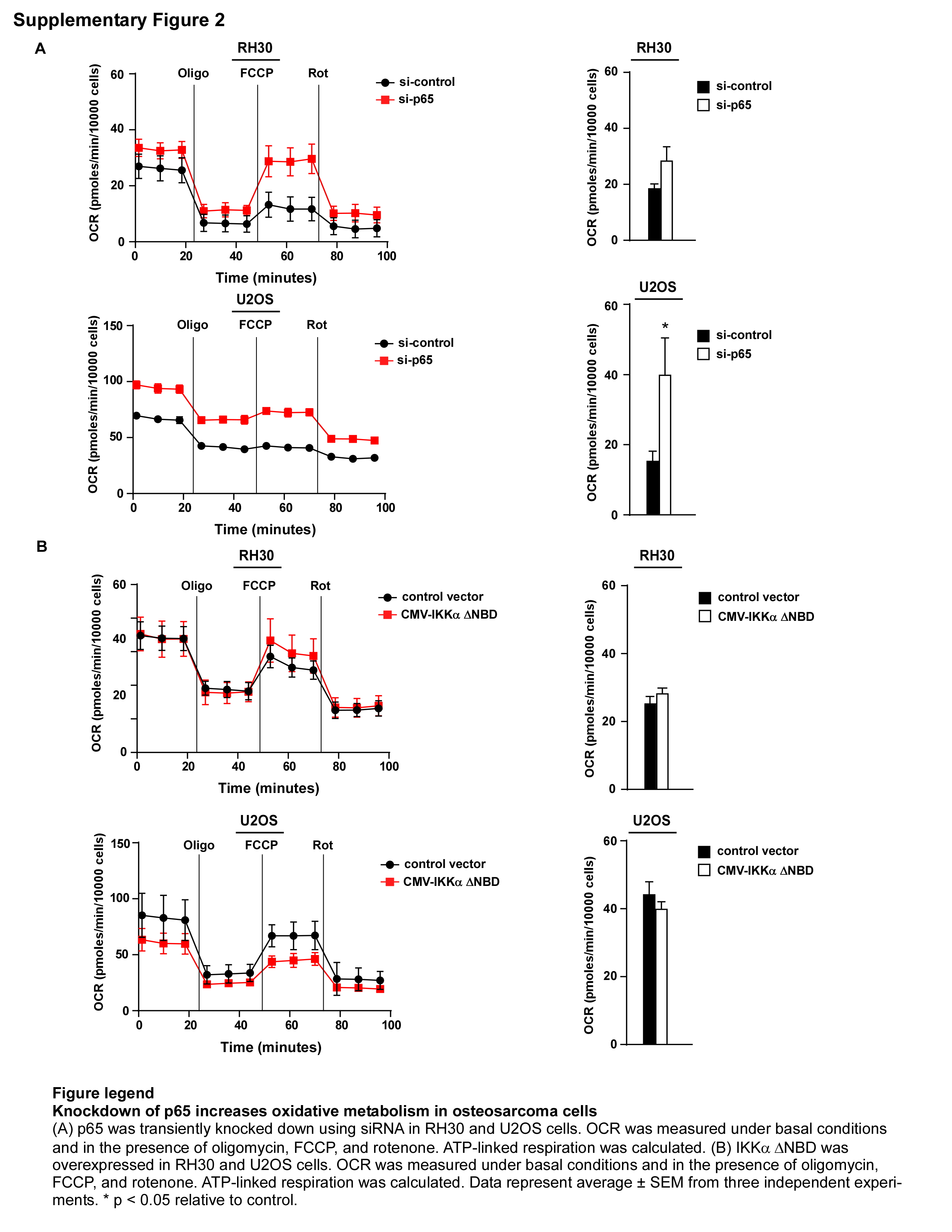

Supplement: Supplementary file 2 [file Image_2.JPEG]

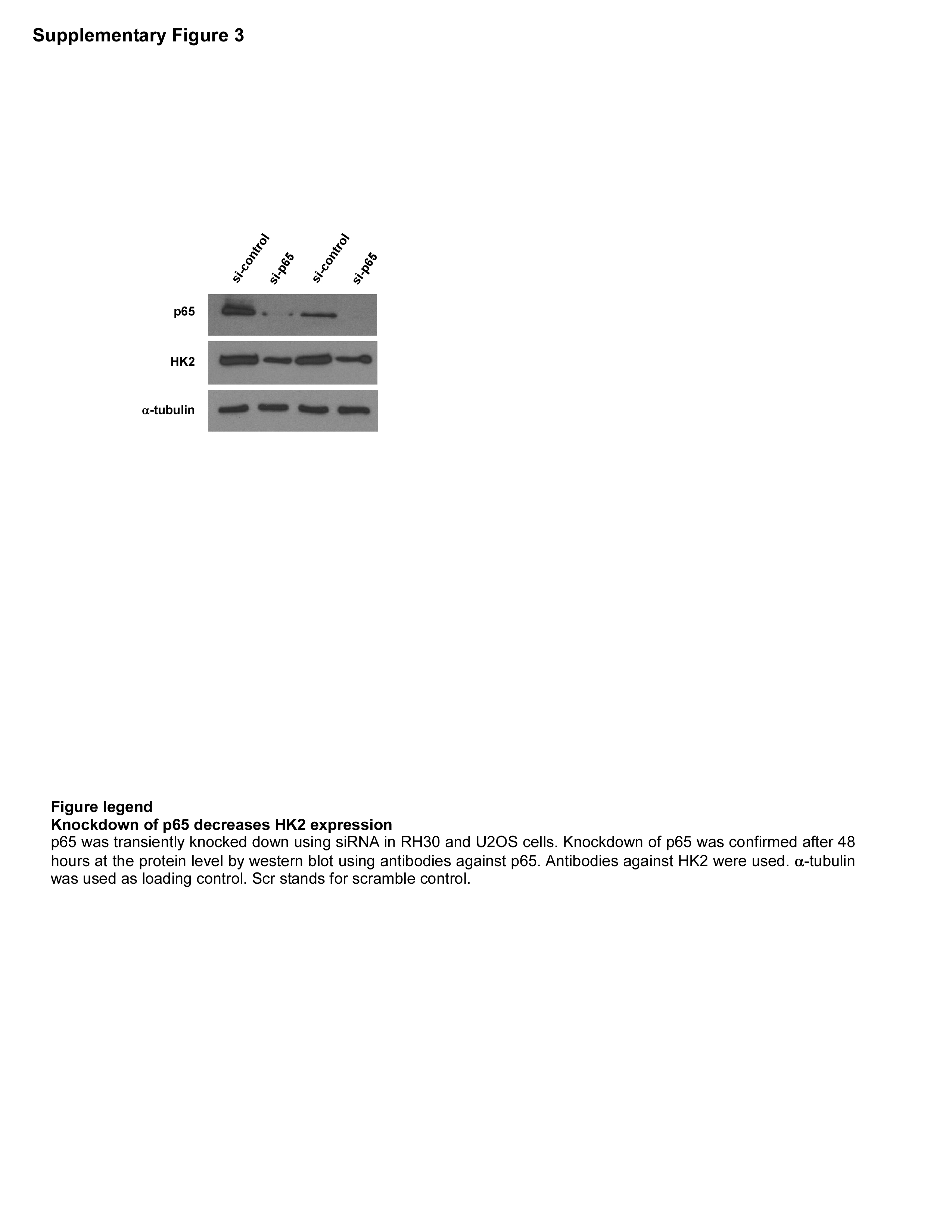

Supplement: Supplementary file 3 [file Image_3.JPEG]

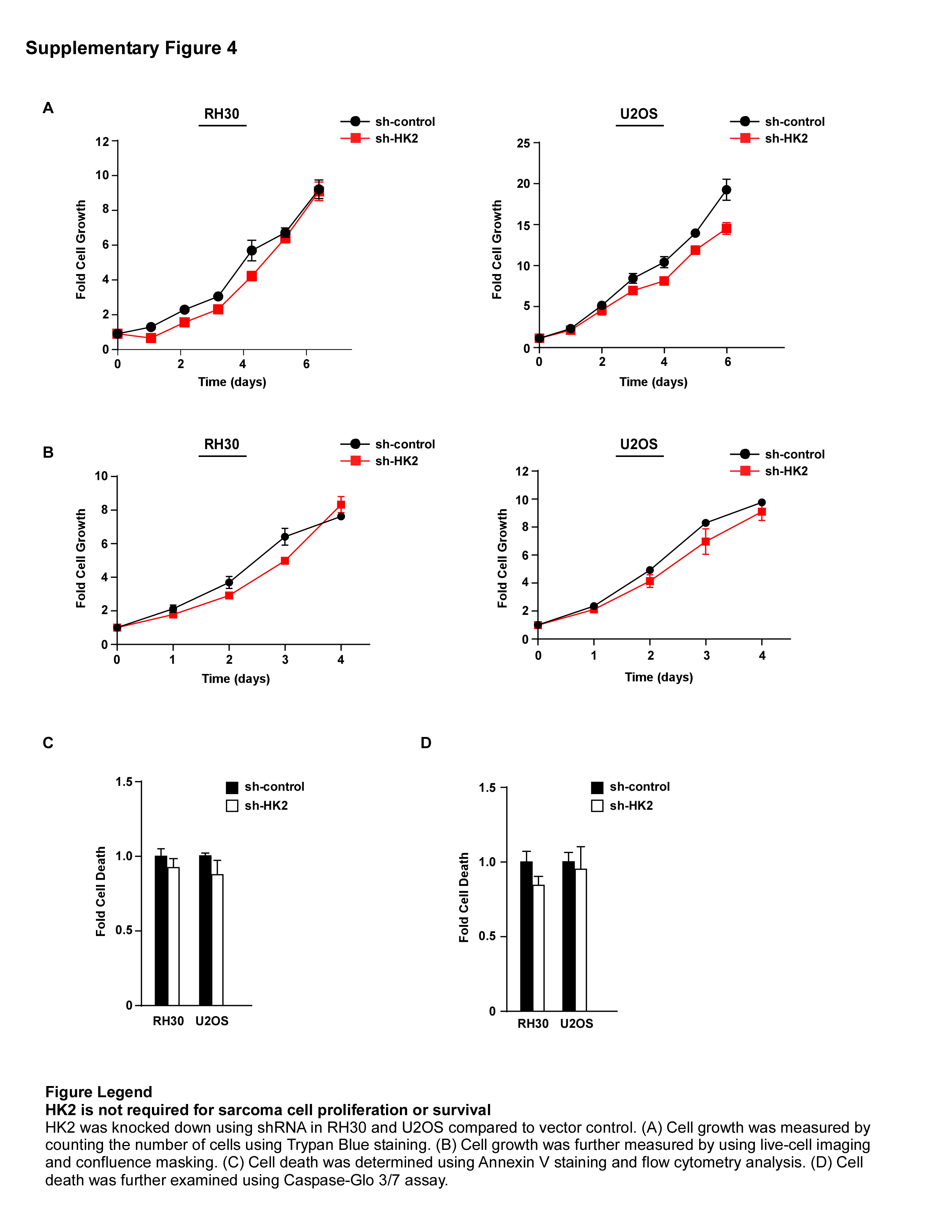

Supplement: Supplementary file 4 [file Image_4.JPEG]
